# Regional differences of outpatient physician supply as a theoretical economic and empirical generalized linear model

**DOI:** 10.1186/s12960-015-0088-1

**Published:** 2015-11-17

**Authors:** Stefan Scholz, Johann-Matthias Graf von der Schulenburg, Wolfgang Greiner

**Affiliations:** Department of Health Economics and Management, Faculty of Public Health, University of Bielefeld, Universitätsstr. 25, 33615 Bielefeld, Germany; Institut für Versicherungsbetriebslehre, Leibniz Universität Hannover, Otto-Brenner-Straße 1, 30159 Hannover, Germany

**Keywords:** Physician supply, Physician density, Spatial analysis, Regional analysis

## Abstract

**Background:**

Regional differences in physician supply can be found in many health care systems, regardless of their organizational and financial structure. A theoretical model is developed for the physicians’ decision on office allocation, covering demand-side factors and a consumption time function.

**Methods:**

To test the propositions following the theoretical model, generalized linear models were estimated to explain differences in 412 German districts. Various factors found in the literature were included to control for physicians’ regional preferences.

**Results:**

Evidence in favor of the first three propositions of the theoretical model could be found. Specialists show a stronger association to higher populated districts than GPs. Although indicators for regional preferences are significantly correlated with physician density, their coefficients are not as high as population density.

**Conclusions:**

If regional disparities should be addressed by political actions, the focus should be to counteract those parameters representing physicians’ preferences in over- and undersupplied regions.

**Electronic supplementary material:**

The online version of this article (doi:10.1186/s12960-015-0088-1) contains supplementary material, which is available to authorized users.

## Introduction

Regional differences in the physician-population ratio are a reality in most countries, even in those with a social health insurance or a national health service [1]. Germany is no exception, and there is an ongoing debate in whether physician supply (i.e., the number of practicing physicians) fits the needs (i.e., number and morbidity of inhabitants) of the population and how differences in regional physician supply can be overcome (e.g., Ozegowski and Sundmacher [2,3]; Kistemann and Schröer [4]). The permission to open a practice within a certain area is regulated by the 17 Associations of Statutory Health Insurance Physicians—Kassenärztliche Vereinigung (ASHIPs) since 1993 within each of the 16 states of Germany (North Rhine-Westphalia is covered by two ASHIPs). For each of the 412 districts in Germany (which represent the second smallest administrative level), a physician supply rate is calculated as the percentage of current physicians per inhabitants in relation to the physicians in the year 1990 (specialists) or 1995 (physicians). A time constant ratio between physicians and population therefore corresponds to a supply rate of 100%. Physicians are only allowed to open a new practice location within a district whose supply rate lies beneath 110%. Following a new legislation (GKV-Versorgungsstrukturgesetz (GKV-VStG)) in 2012, this needs-based planning procedure is not only based on the absolute number of persons living in the planning area but supplemented by its demographic structure (share of persons over 65 years) to calculate the number of physicians allowed to practice in this area. Nevertheless, there are still significant, historically grown regional differences in physician supply. The average supply rate in Germany was 126.5% over all groups of physicians in 2010 with a minimum of 93.0% for general practitioners (GPs) in Saxony-Anhalt and a maximum of 266.2% for surgeons in Mecklenburg Western Pomerania [5].

As mentioned above, there is an ongoing discussion how to equalize the physician-population ratio between the different districts, and some federal state governments have imposed measures to give incentives to physicians to move to so-called underserviced areas (supply rate <75%). Thereby, the legal actions on the planning procedure as well as the incentives focus on the supply side and were taken without any theoretical and empirical basis [6,7]. It is surprising that the demand-side factors play a minor role, since in reality they may actually play a role as the studies from Newhouse [8], Krishnan [9], and Russo et al. [10] have shown. The need for physicians’ services is dependent on the morbidity of the population, which is known to show large regional differences [11]. In addition, it may be more burdensome in rural areas to visit a physician’s practice due to the lack of public transportation and longer traveling times. In this case, differences in physician-population ratio can be explained by lower patient-population ratio. Incentives for physicians to move to areas with a low physician-population ratio would increase overall allocative efficiency.

So far, a variety of research has focused on different aspects of physicians’ behavior, such as physician-induced demand, e.g., Busato and Kunzi [12], Farley [13], Hemenway and Fallon [14], Jaegher and Jegers [15], and Rice and Labelle [16], or the influence of competition on practice patterns, e.g., Davis et al. [17], Folland and Stano [18], or Reinhardt [19]. However, there are only a few publications directly examining the process of practice-location allocation. Wennberg et al. [20] developed a conceptual framework on how planning for physician supply should be implemented but lack a theoretical model to explain regional differences in physician supply. On an empirical basis, Cooper et al. [21] showed on a macro-level that economic development is positively associated with physician supply as well as with related utilization. The studies from Ricketts and Randolph [22], Ricketts [23], and Ricketts [24] analyzed what factors of departure- and destination-district as well as physician characteristics influence the decision of practice location by moving physicians.

Unfortunately, as these studies are set in the U.S., their transferability to other countries is limited, and more generally, it remains unanswered whether these implications also apply for small areas within a country or for all physician and not just for moving physicians.

The aim of the present paper is to provide a theoretical framework to model physician behavior with regard to practice-location allocation which is transferable to other health care systems. The model is examining the influence of various factors on the regional distribution of medical practices as a basis to systematically tackle the problem of regional differences in physician supply. The model leads to some hypotheses as a basis for the empirical verification as the second part of the study. German data is used for this verification, and the combined results of the theoretical and empirical model are discussed in the rear section of the paper.

## Theoretical model

### General motivation and model layout

In theory, the spatial differences of physicians can be explained by demand-side as well as by supply-side factors. The regional distribution of physicians is usually seen as a market failure due to physician-induced demand (see Richardson and Peacock [25], van Dijk et al. [26]) calling for governmental intervention. Our model does not assume that physicians are able to induce demand for their services, so that the physician density in a given region is determined by the patients’ demand and the regional preferences of the physician (see for physician regional preferences Matsumoto et al. [27]). A more detailed overview of supply-side factors from the literature is given and examined in the last part of this section.

The theoretical model has four parts. The first part presents a model of the demand-side. It is a simple model of a representative individual who demands services by general practitioners (family doctors) and specialists, as we are also interested in the composition of the physician in urban and rural areas. In the second part, a consumption time function is introduced. Our hypothesis is that search, traveling, and waiting time play an important role for the consumer for his or her decision to consult a doctor. The higher the time cost to consume a doctor, the lower is the demand. With this approach, we incorporate a well-known argument already raised by the seminar article of Phelps and Newhouse [28].

The third part presents a model for physicians deciding to settle in a certain region. We assume that physicians like other professionals are interested in income which is the revenues deducted by the cost of running the office. In the fourth and final part, we derive the regional equilibrium to determine the physician-population ratio in urban and rural areas. In this part, we also introduce a factor covering the regional preferences of physicians. We assume that a physician who settles in a more preferred region is willing to sacrifice for this part of his or her income. A regional equilibrium is reached, if every physician belonging to a certain specialty earns the same income weighted by the regional preference factor.

In our model, we assume two regions, an urban (u) and a rural one (r). Both regions have the same population (*n* = *n*_u_ = *n*_r_) but differ in their size measured in square miles or square kilometers *m*. The size of the rural region is *m*_r_ and of the urban area *m*_u_ (*m*_u_ < *m*_r_). Thus, the population density *n*/*m* is higher in u than in r. The regional physician density is GP/*m* and specialist (SP)/*m*, where GP and SP are the number of practitioners and specialists, respectively. The number of physicians per capita is GP/*n* and SP/*n*.

It is assumed that there is no regional difference in morbidity or demographics, with all consumers and physicians having the same preferences and physicians—as mentioned above—not being able to induce demand. There are no quality differences between physicians, and the services provided are homogeneous.

### Demand for physicians’ services

We consider a representative individual that has a fixed income of *y*, which is spent solely for consumption. The expenditure for consumption—except physician services—is *Y*. The payment for health insurance is payroll tax on income *y* at a tax rate *b. A* and *S* are the services of general practitioners and specialists, respectively. *p* is the price for those services. That means *p*_*A*_ is the price for one unit of services by a GP. *p*_*S*_ is the price for consulting a SP. *á* represents the co-insurance rate, so that the patient has out-of-pocket payments of *áp*_*A*_ or *áp*_*S*_ for a service unit of a GP or a SP. Thus, the monetary constraint for consumption is given by

1 $$ y = Y + b\ y + \acute{a}\kern0.5em {p}_A\ A + \acute{a}\kern0.5em {p}_S\ S $$

The consumer has also a time constraint with his or her time budget being *l*. This is distributed on consumption time *L* and for the time consulting a doctor. The time coefficients *t*_*i*_ (with *i* = *A*,*S*) are the time needed to see a physician, including travel time, waiting time, and consultation time.

2 $$ l = L + {t}_A\ A + {t}_S\ S $$

To sum up, the consumer spends his or her money and time either for physician services or for other consumption. The resources (money and time) taken for other consumptions are expressed by

3 $$ Z=Y + \varrho L $$

where *ϱ* denotes the individual’s opportunity cost of time. In a perfect world with flexible work time, it would correspond to the wage rate (see, for example, Frank [29]).

To finalize the demand model, it is assumed that the individual maximizes a utility function. To receive concrete results, this function is specified. We assume a Cobb-Douglas-type utility function:

4 $$ U={A}^a{S}^s{Z}^z,\ s>a $$

This type of function has plausible properties as described in the literature. *s* > *a* means that specialists’ activities are preferred over GPs’ activities, but GPs’ activities cannot be fully substituted by specialists’ activities and vice versa. Obviously, both types of services are needed, but a partial substitution is possible. Our assumption that patients may substitute one physician type for another if needed is in line with general observations and empirical studies. McLeod [30] showed, with data from the Canadian Community Health Survey and the Ontario Health Insurance Program, that a shortage in the supply of one physician type can result in an increase in the use of other physician types.

Our model does not cover the fact that the demand for specialists’ services can be the result of referrals of general practitioners. In some countries, like Germany, Austria, and Switzerland, patients may consult a specialist directly without a referral from a family doctor. In other countries, like Great Britain, a family doctor has to be consulted first before going to a specialist. In some countries, in addition, specialists do only work in hospitals. Gächter et al. [31] have shown for Austria that referrals from GPs to SPs play a role and that GPs and SPs collaborate with each other, so that both markets are interrelated.

Inserting the monetary constraint (1) and the time constraint (2) in (3) leads to

5 $$ 0 = Z\ \hbox{--}\ y + b\ y + \acute{a}\ {p}_A\ A + \acute{a}\ {p}_S\ S - \varrho\ l + \varrho\ {t}_A\ A + \varrho\ {t}_S\ S $$

The (representative) individual maximizes his or her utility function (6) subject to (5), whereby *Q* is the maximization operator and *λ* a Lagrange multiplier, which is used to maximize an objective function under constraints.

6 $$ \max\ Q = {A}^a{S}^s{Z}^z + \lambda \left[Z\ \hbox{--}\ y + by + \acute{a}{p}_AA + \acute{a}{p}_SS - \varrho l + \varrho {t}_AA + \varrho {t}_SS\right] $$

Differentiating (6) with respect to *A*, *S*, *Z*, and *λ* leads to the first-order conditions for an optimal consumption plan. If we divide these conditions pairwise, we obtain

7 $$ \frac{S}{A} = \frac{\left(\acute{a}{p}_A+\varrho\ {t}_A\right)}{\left(\acute{a}{p}_S+\varrho\ {t}_S\right)}\ \frac{s}{a} $$

8 $$ \frac{Z}{S}=\left(\acute{a}{p}_S+\varrho {t}_S\right)\ \frac{z}{s} $$

9 $$ \frac{Z}{A}=\left(\acute{a}{p}_A+\varrho {t}_A\right)\frac{z}{a} $$

As the model is constructed, the relative demand for services of GPs and SPs and the demand for other consumptions depend on the insurance coverage, the price of other consumptions, the time needed to consume physician services, and the preferences expressed by the coefficients in the utility function.

If, for instance, the time needed to consume specialist services, *t*_*s*_, is higher or the preference for those services is lower, the demand for services of general practitioners *A* will be higher relative to *S* and *Z* in the utility-maximizing consumption plan.

In the next step, we solve the equations (7) to (9) for *S* and *Z*, respectively. This allows us to replace *S* and *Z* in (5) by (7) to (9), so that only the variable *A* is left and *S* and *Z* have been replaced. We obtain

10 $$ \left(\acute{a}{p}_A+\varrho {t}_A\right)\ \frac{z}{a}\ A-y\left(1-b\right)-l+\left(\acute{a}{p}_A+\varrho {t}_A\right)A+\left(\acute{a}{p}_S+\varrho {t}_S\right)\ \frac{\left(\acute{a}{p}_A+\varrho {t}_A\right)}{\left(\acute{a}{p}_S+\varrho {t}_S\right)}\ \frac{s}{a}\ A=0. $$

This leads after reorganization to

11 $$ A = \frac{a\ \left(y\left(1-b\right)+\varrho l\right)}{\left(\acute{a}{p}_A+\varrho {t}_A\right)\left(a+z+s\right)}. $$

In the same way, we can derive from (7) to (10) the equation for *S*:

12 $$ S = \frac{s\ \left(y\left(1-b\right)+\varrho l\right)}{\left(\acute{a}{p}_S+\varrho {t}_S\right)\left(a+z+s\right)}. $$

By multiplying the number of inhabitants in each region with the demand for physician services, we receive the demand for the whole region, i.e., *nA*_u_, *nA*_r_, *nS*_u_, and *nS*_r_. This demand is dependent on the number of inhabitants (which is assumed to be *n* and the same in each region); the preferences expressed by *a*, *s*, and *z*; the net income (1 − *b*)*y*, the monetary cost *áp*_*A*_ (which is larger than 0 if the co-insurance *á* is positive); and the time cost *ϱt*_*S*_ to consume the services (which are different in the regions due to difference in traveling time). If we divide *nA*_u_, *nA*_r_, *nS*_u_, and *nS*_r_ by the number of physicians, e.g., GP_u_, GP_r_, SP_u_, and SP_r_, we receive the average number of services provided by each physician.

### Time cost

The time cost to consult a physician includes travel time, waiting time, and time of consultation. Traveling time and by this time cost decrease with the average regional distance between physicians’ practices. Whereas in urban areas the nearest doctor’s office can be reached in just a few minutes, travel times (hence time cost) in rural areas are more considerable.

Following this argument, we assume the following time cost function:

13 $$ {t}_{A_i} = {\left(\frac{{\mathrm{GP}}_i}{m_i}\right)}^{-{q}_A},\ \mathrm{with}\ i = \left\{\mathrm{u},\mathrm{r}\right\};\ 0 < {q}_j < 1;\ {q}_S > {q}_A $$

14 $$ {t}_{S_i} = {\left(\frac{{\mathrm{SP}}_i}{m_i}\right)}^{-{q}_S} $$

where GP_*i*_ stands for the number of general practitioners, SP_*i*_ for the number of specialists, and *i* for the region. *m*_i_ measures the size of the region. Although the population is assumed to be the same in both regions, the rural region is larger than the urban one. *q*_*A*_ and *q*_*S*_ are the time cost coefficients. If *q*_*j*_ (*j* = *A*,*S*) is 0, the time cost does not vary with the physician density. If *q*_*j*_ is 1, the time cost varies proportional with the physician density. We assume *q*_*j*_ to be between 0 and 1.

The explanation for the chosen time cost equation is as follows: The time cost is dependent on various factors, but also on the regional physician density, because the average travel time increases for the patients if there are fewer physician offices in the region. If the number of physicians goes up in a certain region, the time cost will decrease and *q* is assumed to be smaller than 1, because the traveling time is only one component of the time needed to visit a physician’s office. The patient also has to invest time to search for the right physician, needs the waiting time in the office, and the time for the treatment itself. *q* smaller than 1 also implies that the demand for physician services does not grow faster than the number of physicians, i.e., the time-cost-physician elasticity is smaller than 1.

It is plausible that it takes more time to find an appropriate specialist, due to the increasing variety of specialists. Because the scarcity of certain specialists in large areas also increases travel time, we assume *q*_*A*_ < *q*_*S*_.

### Physician behavior

The third part of the model specifies physician behavior. For simplicity, we assume that physicians have some regional preferences, which we will introduce in the next section, but are interested in their income. So physicians move to that region where they receive a higher income.

The prices for the physicians’ services are the same in both regions but differ between the types of physicians, e.g., they are *p*_*A*_ and p_S_. Assuming constant marginal cost and that all physicians have the same number of patients in a given region, the income of a GP and a SP in a rural or urban region is as follows, where *Y*_*Ai*_ and *Y*_*Si*_ with *i* = u, r are the income of a physician in the urban and the rural region, respectively, and *c*_*A*_ and *c*_*S*_ are the marginal cost for one service unit of a GP or a SP, respectively:

15 $$ {Y}_{A_i}=\left({p}_A-{c}_A\right)\ {A}_in/{\mathrm{GP}}_i\ \mathrm{with}\ i=\mathrm{u},\mathrm{r} $$

16 $$ {Y}_{S_i}=\left({p}_S-{c}_S\right)\ {S}_in/{\mathrm{GP}}_i\ \mathrm{with}\ i=\mathrm{u},\mathrm{r} $$

with *j* = {GP, SP}, *i* = {u, r}.

If physicians are free to choose where to open their practice, in a state of equilibrium, physicians’ incomes are the same in both regions:

17 $$ {Y}_{A_{\mathrm{r}}} = w\ {Y}_{A_{\mathrm{u}}},\ w\ \ge\ 1 $$

and

18 $$ {Y}_{S_{\mathrm{r}}} = w\ {Y}_{S_{\mathrm{u}}},\ w\ \ge\ 1 $$

*w* expresses the regional preference of physicians. If *w* is 1, physicians have no regional preferences. If it is greater than 1, they prefer urban areas, i.e., they are willing to sacrifice part of their income to live in a preferred region. Physicians are producers and as well as consumers, and they choose to go to places for many reasons. One reason is certainly to have enough patients. But as a consumer, other reasons are important, like attractiveness, cost of living, and personal tasks.

It is certainly a rough simplification to assume that urban areas are always more attractive for physicians than rural ones. For instance, Matsumoto et al. [23] have shown in a study on Japanese physicians that the attractiveness of a municipality depends on the amenities of urban life which seems to be more highly correlated with the number of “daytime population” and “service industry population” than total population. Erus and Bilir [32] have published recently results from a Turkish study, which shows that after the regulation was lifted that young doctors have to go first in underserved regions; socio-economic conditions of a region became a significant determinant of availability of specialists. So it is important to detect the factors or proxies for the attractiveness of a region.

### Regional equilibrium

In the next, final part of the model, we bring together the three previous parts of the model and derive some conclusions. We insert (11) to (16) in (17) and (18) and receive for *á* = 0 the following equations:

19 $$ \frac{{\mathrm{GP}}_{\mathrm{u}}}{{\mathrm{GP}}_{\mathrm{r}}}={\left(\frac{m_{\mathrm{r}}}{m_{\mathrm{u}}}\right)}^{\frac{q_A}{1-{q}_A}}\ {w}^{\frac{1}{1-{q}_A}} $$

20 $$ \frac{{\mathrm{SP}}_{\mathrm{u}}}{{\mathrm{SP}}_{\mathrm{r}}}={\left(\frac{m_{\mathrm{r}}}{m_{\mathrm{u}}}\right)}^{\frac{q_S}{1-{q}_S}}\ {w}^{\frac{1}{1-{q}_S}} $$

(19) and (20) show that the number of physicians and hence the physician density is larger in urban than in rural areas, because the right-hand side is greater than 1. The urban-rural discrepancy increases if the regional preference for urban areas increases.

Dividing (20) by (19) yields to

21 $$ \frac{{\mathrm{SP}}_{\mathrm{u}}}{{\mathrm{GP}}_{\mathrm{u}}}=\frac{{\mathrm{SP}}_{\mathrm{r}}}{{\mathrm{GP}}_{\mathrm{r}}}\ {\left(\frac{m_{\mathrm{r}}}{m_{\mathrm{u}}}\right)}^{\frac{q_S-{q}_A}{\left(1-{q}_A\right)\left(1-{q}_S\right)}} $$

Obviously, the specialist-general practitioner relationship is higher in urban than in rural areas if *q*_*A*_ < *q*_*s*_.

Our model leads to four hypotheses:

1. Regional preferences of physicians lead to differences in regional physician-population ratio, even if physicians are not able to induce demand for their services. In our model, the preferences of physicians for urban areas are reflected by the parameter *w*. We will introduce a number of proxies for the attractiveness of a region in our empirical analysis.
2. If physicians have no regional preference, it is also plausible that the physician-population ratio for each specialist group is higher in regions with a high-population density than in rural areas. If traveling and waiting time plays a role for the demand for physician services, and these time costs are high in a given region because the physician-population ratio is low, the demand will increase, if the physician-population ratio increases.
3. Not only the absolute number of specialists but also the GP-specialist ratio is higher in urban than in rural areas, if search and travel time costs are higher for specialists. As the two first propositions derived from the model are straightforward, the third proposition needs to be explained. The reason is the heterogeneity of specialists compared with general practitioners. The more different types of specialists exist, the higher are the time costs to find the right doctor for the patient’s specific health problem. An increase in the number of physicians will increase the demand for their services due to the reduced time cost. But this effect is larger for SPs than for GPs.
4. The higher the level of insurance coverage of physician services or the lower the co-payments, the higher the regional inequality of outpatient care. This can be derived directly from our model. If, for instance, *á* is set equal to 1 instead of 0, the monetary cost increases. By this, the relative importance of the time cost decreases.

Hypotheses 1 and 2 make clear that it is an empirical question whether differences in physician-population ratio are really a sign of market failure. The question is whether and to which extent demand differences or regional preferences of the physicians are associated with the differences in physician-population ratio. One factor causing demand differences is the time cost associated with medical treatment. Other factors are morbidity differences. While the regional physician-population ratio and the size of a region are known, the challenge of an empirical study will be to detect the right proxies for the demand for medical services and the attractiveness of a region.

The model is simple and, not surprisingly, it has a number of limitations. One limitation of the model is the assumption that physicians are income maximizers. If physicians behave in a purely altruistic manner, then the model does not correctly describe physician behavior. However, our hypothesis that monetary incentives are one of the main drivers for the decision of setting up a practice is confirmed by Günther et al. [33]. In a survey conducted among 14 939 German practicing non-postgraduate physicians, the authors used a discrete-choice experiment to weight the attributes of hypothetical locations for practices. Income was weighted with the highest utility weight.

### Physician preferences and supplementing factors

However, other parameters also play a role in the decision for a certain location, and it is important for the empirical validation of our model to find appropriate proxies not only for demand but also for the supply side from the literature. Also in the study of Günther et al. [33], the authors found the attributes *professional cooperation*, *leisure activities*, *career opportunities of the partner*, and *availability of child care* followed the utility weight of income in descending order from qualitative interviews. Thereby, the last three attributes were equalized by the driving distance. This survey also included a questionnaire asking physicians about the importance of 18 items on their decision for a practice location. Using factor and regression analysis on the results of the questionnaire, Roick et al. [34] found financial incentives to be less important than a *positive environment for the family* and *occupational duties*. On the other hand, *possibilities for cooperation*, *conditions of service*, and *quality of life* in their living area in descending order are of lower relevance.

The study from Kazanjian and Pagliccia [35] used a very similar methodological framework as Roick et al. [34] to retrospectively analyze Canadian physicians’ choice of practice location in 1989. The authors focused on physicians’ satisfaction with the location of their current practice on the basis of 41 items as well as on persons and events influencing the decision for this location and thereby differentiate between physicians practicing in rural and urban areas. Physicians’ spouses, the desire to raise a family in an environment similar to that of their own childhood, peers, and friends have the highest influence on the location of physicians’ practices. Nevertheless, income and other location factors are only part of the equation when analyzing satisfaction with the current location of practice and therefore do not allow any conclusions to be drawn between them and the primary choice of residence.

An empirical model from Breyer et al. [36] analyzed the determinants of utilizing physician services and the supply side in Germany. It showed that the determinants of physician supply are explained by per capita expenditure for physician services, population density, gross domestic product (GDP) of the region, hospital beds per 1000 inhabitants, and overnight stays in hotels. The last two parameters are assumed to cover the distance to the physicians’ training location, i.e., university hospital, and the cultural and recreational appeal of a region. In the regression analysis, only expenditure for physicians, hospital bed density, and overnight stays in hospitals showed a significant association with the physician density.

A comprehensive analysis has also been conducted by Kistemann and Schröer [4]. In this study, the target and actual numbers of GPs and different specialists are compared in a German district on the postal-code level as a function of the portion of inhabitants of high socioeconomic status. In an additional postal survey, physicians were asked about the importance of economic and personal factors on the decision for their current practice location. The authors found an equal number of non-economic and economic factors being rated “important”.

As these publications reveal, economic incentives, measured either directly over the expenditure for physician services as in Breyer et al. [36] or indirectly over the population density as in our model, are not exclusively responsible for physicians’ decision regarding the location of their practice. Instead, the professional environment for the physician, a labor market for the physicians’ spouses, the accessibility of cultural and recreational activities, and the attractiveness of a district are contributing factors to this decision. The following empirical analysis seeks to examine to what extent the propositions of our model can be found in German outpatient care and which other spatial factors are associated with the number of GPs and specialists.

## Empirical evidence

### Data

The data for the analysis were obtained using datasets from different sources. The absolute number of physicians of different disciplines has been made available by the federal ASHIP for the year 2010 for 412 German districts. The independent variables on the district level have been combined with respect to the findings in the literature under the topics *population*, *morbidity*, and *financial incentives* for the demand-side factors and *health care system*, *culture*, *labor/economy*, *attractiveness*, and *infrastructure* for the regional preferences of the physicians, i.e., the supply side. *Population* parameters correspond to the parameters used in the German governmental planning procedure and include the overall number of inhabitants in the year 2010, which were available by age and sex from the German Federal Statistical Office [37]. The old-age dependency ratio, which was calculated in accordance to Rawland [38] using these data, represents the *morbidity* of the population in combination with the sex-specific life expectancy and the overall mortality of a district. To capture *financial incentives* of a district, the mean household income and the share of privately insured patients were used [39]. Private health care insurance in Germany follows a different reimbursement scheme offering physicians a higher level of remuneration.

To contro*l for the general structure of the health care system*, the respective number of hospital and nursing home beds as well as the presence of a university hospital have been included to account for the possibility of cooperation with other doctors. The number of middle-order (defined as cities providing specialist doctors, shopping malls, cinemas, hospitals, public swimming pools, and legal counselors) and high-order centers (defined as cities additionally providing special shops, hospitals, and cultural, educational, and administrative institutions) can be seen as variables describing more densely populated areas, thus offering higher cultural variety. Supplemented by the location of state capitals and cities with more than 100 000 inhabitants, these variables are summarized as the *culture* of a district. Covering the labor market for physicians’ spouses, the unemployment rate, the rate of highly qualified workers, and the gross domestic product (GDP) per capita were included under the term *labor market and economy* of a district. The last category of independent variables contains the *attractiveness* of a district, measured by the number of guest-nights in tourist enterprises per capita, the difference in persons having moved permanently to and from the district in the last 5 and 10 years, respectively, and the intensity of exchange of real estate. Finally, the average travel time to the nearest airport, high-speed train station, middle-order and high-order centers for 2010 were used as *infrastructure* variables for each district to depict not only provision of cultural variety in more densely populated areas by the physicians but also their accessibility from inside or outside the district as patients might also consult physicians in other districts besides their residence. Except for the population parameters, the data were extracted from the INKAR dataset of the Federal Institute for Research on Building, Urban Affairs and Spatial Development [40] for the year 2010, unless stated otherwise. All variables used in the analyses are shown in Table 1. Variables which are not shown in the results did not contribute to the model significantly. In addition, data from the Association of German University Hospitals was used for identifying districts with university hospitals [41]. As only secondary data was used in the analyses, no ethical approval or patient consent was necessary.

**Table 1:** **Dependent and independent variables on district level** **(** ***n*** **= 412)**

**Topic**	**Metric variables**	**Mean**	**Standard deviation**	**Minimum**	**Maximum**
Dependent	Density of GPs (per 10 000 inhabitants)	6.32	0.99	1.52	12.58
	Density of specialists (per 10 000 inhabitants)	3.14	1.35	0.51	8.99
	Ratio GPs/specialists	2.30	0.99	0.75	12.80
Demand/need factors					
Population	Population density (per km^2^)	518.68	674.91	37.09	4355.28
Morbidity	Old-age dependency ratio	32.14	4.22	22.03	45.53
	Life-expectancy women (from 60 years)	25.08	0.63	23.10	27.10
	Life-expectancy men (from 60 years)	21.56	0.94	19.40	24.60
	Mortality (deaths per 1000 inhabitants)	10.91	1.59	6.90	15.40
Financial incentives	Household income (in €, per month)	1548.93	199.31	1157.90	2585.00
	Rate of privately insured persons (%)	13.46	4.32	3.53	27.00
Control factors					
Health care system	No. of hospital beds (per 10 000 inhabitants)	64.49	38.70	0.00	215.90
	No. of nursing home beds (per 10 000 inhabitants)	108.94	28.83	47.10	256.60
Cultural	No. of middle-order centers	2.24	2.16	0.00	11.00
	No. of high-order centers	0.39	0.56	0.00	4.00
Labor/economy	Unemployment rate (%)	7.41	3.31	1.90	17.40
	Rate of highly qualified workers (%)	8.23	3.80	3.00	26.50
	GDP per capita (in €1000)	27.58	10.24	13.20	83.60
Attractiveness	Touristic attractiveness	5.27	7.56	0.00	90.60
	Building area attractiveness	125.47	117.34	0.00	1031.80
	Migration balance (last 10 years)	4.54	46.13	−171.40	100.30
	Migration balance (last 5 years)	−3.98	21.85	−69.80	61.80
Infrastructure	Travel time to airport	54.41	24.11	7.60	161.50
	Travel time high-speed train station	22.36	14.42	0.00	61.60
	Travel time middle-order center	8.26	6.40	0.00	36.60
	Travel time high-order center	26.56	17.83	0.00	76.20
	**Binary variables**	**Frequency**		**Percentage**	
Cultural	State capital	16/412		3.88	
	City >100 000 inhabitants	68/412		16.50	
Health care system	University hospital	33/412		8.01	
Infrastructure	District in former East Germany	86/412		20.87	
	Urban district	206/412		50.00	

### Analytical method

Three separate models were estimated using the density of GPs per 10 000 inhabitants, the density of general specialists^a^ per 10 000 inhabitants, and the ratio between the absolute number of GPs divided by the absolute number of general specialists as dependent variables. As the number of physicians can be seen as numeric count data taking no zero values, a generalized linear model (GLM) with log-link using a zero-truncated negative-binomial distribution was estimated using the logarithmized number of 10 000 inhabitants as an offset, thus estimating physician density [42]. By considering varying intercepts on the ASHIP level, the model accounts for possible correlations in the number of physicians between districts lying in the same ASHIP region (the value for each ASHIP can be found in Additional file 1). The first two models were also carried out using physician and population density under the assumption of a normally distributed physician density. As the physician density was positively skewed and showed some outliers, the use of a GLM model was preferred. Furthermore, Poisson regression was omitted because of over-dispersed data. The model takes the following form with *g*() being a log-link function and *α*_*i*_ and *α*_*k*_ representing the intercepts of the district and the corresponding ASHIP, respectively. The logarithm of the inhabitants serves as the offset.

$$ g\left({y}_{ik}\right) = {\alpha}_k+{\alpha}_i + \boldsymbol{X}\boldsymbol{\beta } +\mathrm{offset}\left( \log \left({\mathrm{inhabitants}}_i\right)\right) + \varepsilon $$

Where applicable, the independent variables were transformed to *z*-scores by subtracting the mean and subsequently dividing by two standard deviations to allow a coherent interpretation between metric and binary variables [43]. Therefore, the intercept needs to be interpreted as a predictive mean value which correlates to the imputation of the mean values of the independent variables, and the coefficients of *z*-scored variables need to be interpreted as the effect of an increase or decrease of the variable by two standard deviations. To retain comparability between the three models, variables showing significance at the 0.05 level in at least one of the models are kept in all the models. The analyses were started by including all explanatory variables, with interactions being considered where reasonable. To receive the final model, coefficients and their corresponding interactions were excluded from the model in a backward stepwise manner, starting with excluding the variable with the highest *P* value. Regression diagnostics are provided in Additional file 1. All analyses were performed with R version 3.0.1 using the package *gamlss* [44,45].

## Results

### General practitioners

The regression model for GPs estimates a logarithmized (log) physician density of 1.765 (=5.81 GPs per 10 000 inhabitants) in a district in the ASHIP of Brandenburg with a mean population density, household income, share of privately insured persons, number of hospital beds, number of middle- and high-order centers, having no state capital or city having more than 100 000 inhabitants, mean rates of highly qualified workers and unemployment, a mean migration balance of the last 5 years, and touristic attractiveness as well as travel time to the nearest airport and middle-order center,.

As can be seen from Table 2, population density alone is significantly negative associated with a decrease of −0.136 in the log physician density in the GP model. Nevertheless, population density is strongly interacting with the presence of a city with more than 100 000 inhabitants, balancing out the negative association between population and GP density to some extent (to −0.037). The strongest positive association with GP density can be found for the presence of a state capital in one district (0.127) followed by the number of hospital beds with 0.073. The coefficients of the travel time to the nearest airport, the touristic attractiveness, the share of privately insured persons, and the number of high-order centers are also significantly, positively, and equally strongly associated with a higher GP density. This might also be true for the unemployment rate, although its *P* value lies just over the 0.05 significance level.

**Table 2:** **Results of the three zero-truncated, negative binomial GLMs for GP density, specialists density, and ratio of GPs divided by specialists**

**Topic**	**Coefficients**	**General physicians**		**Specialists**		**Ratio GPs/specialists**	
		**Estimate**	***P*** **value**	**Estimate**	***P*** **value**	**Estimate**	***P*** **value**
	Intercept	1.736	0.000^***^	1.919	0.000^***^	−0.178	0.003^**^
Population	Population density (per km^2^) (*z*-score)	−0.136	0.005^**^	0.349	0.000^***^	−0.453	0.000^***^
Financial incentives	Household income (*z*-score)	0.018	0.255	0.093	0.001^**^	−0.088	0.001^**^
	Share of privately insured persons (*z*-score)	0.042	0.008^**^	0.123	0.000^***^	−0.063	0.033^*^
Health care	No. of hospital beds (*z*-score)	0.073	0.000^***^	0.253	0.000^***^	−0.206	0.000^***^
Cultural	Number of middle-order centers (*z*-score)	−0.005	0.703	−0.074	0.004^**^	0.052	0.038^*^
	Number of high-order centers (*z*-score)	0.036	0.003^**^	0.057	0.022^*^	−0.022	0.336
	City in district (binary)	−0.021	0.476	0.146	0.005^**^	−0.140	0.008^**^
	State capital in district (binary)	0.127	0.000^***^	0.278	0.000^***^	−0.096	0.244
Labor/economy	Rate of highly qualified workers (*z*-score)	0.019	0.264	0.106	0.001^**^	−0.099	0.001^**^
	Unemployment rate (*z*-score)	0.052	0.064	0.139	0.006^**^	−0.073	0.142
Attractiveness	Touristic attractiveness (*z*-score)	0.046	0.000^***^	−0.004	0.861	0.036	0.116
	Migration balance (last 5 years) (*z*-score)	−0.012	0.478	0.072	0.025^*^	−0.113	0.000^***^
Infrastructure	Travel time to the nearest airport (*z*-score)	0.049	0.000^***^	0.021	0.420	0.016	0.548
	Travel time to middle-order center (*z*-score)	−0.064	0.007^**^	−0.177	0.000^***^	0.115	0.004^**^
Interactions	Population density: city in district	0.099	0.044^*^	−0.422	0.000^***^	0.464	0.000^***^
GoF-measures	Sigma (global deviance)	−8.177	0.000^***^	−3.763	0.000^***^	−3.892	0.000^***^
	BIC score first model	3260		4125		3979	
	BIC score final model	3162		3828		3751	

* < 0.05, ** < 0.01, *** < 0.001

The only variable indicating a significant and negative correlation with the GP density is the travel time to the next middle-order center. This means a higher travel time indicates a lower GP density. The effects of the different ASHIPs are statistically significant for 10 of 16 and are all positive, corresponding to the ASHIP of Brandenburg having the lowest GP density. The highest significant coefficient can be found for the ASHIP of Saarland with 0.237. As the ASHIPs are not of primary interest, further details can be found in Additional file 1.

### Specialists

In the model for specialists, the estimated log density of specialists is higher than the estimate for GP density. In a hypothetical district with no city, state capital, and all other variables at the sample mean, the model estimates a log density of 1.919 corresponding to 6.81 specialists per 10 000 inhabitants. In contrast to the GP model, the population density shows the largest (highly significant) association with the specialist density by 0.349. However, this association is again strongly altered by the interactions of population density with cities. With each increase in population density by two standard deviations, the specialist density decreases by 0.422 to −0.073 in a district with a city. Having a city within the district’s borders nevertheless increases the specialist density by 0.146 on its own.

The strongest positive associations of the other variables with the specialist density could be found for the presence of a state capital and the number of hospital beds. A district with a state capital shows 2.18, and an increase of two standard deviations in hospital beds has 1.96 more specialists per 10 000 inhabitants (0.278 and 0.253 increase in log density, respectively). The coefficients of a city in the district (0.146), the unemployment rate (0.139), the share of privately insured (0.123), and the rate of highly qualified workers (0.106) show also significant, strong correlations with the specialist density. Weaker positive effects can be seen for the household income, migration balance, and the number of high-order centers.

Negative associations can be found as well for the number of middle-order centers as for the travel time to the next middle-order center. With −0.177, the first shows the stronger coefficient, whereas with each two standard deviation increases in the number of middle-order centers, the log specialist density is 0.074 lower. The highest difference of a single ASHIP is Berlin with 0.382, although it is only one of three ASHIPs showing a significant association and there also negative coefficients. Again, details are given in Additional file 1.

### GP-specialist ratio

The ratio of GPs divided by the specialists within a district is analyzed in the last model. Thus, negative coefficients are increasing the denominator denoting a higher share of specialists and positive coefficients increasing the numerator denoting a higher share of GPs. With all variables at the sample mean, the ratio of physicians in a rural district in Brandenburg with no city or state capital is estimated to be 0.837 (log = −178). As population density increases by two standard deviations, the log density is reduced by −0.453, implying a higher share of specialists in districts with a higher population density. As with both previous models, the interaction term drastically changes the coefficient of population density in districts with a city. The city variable itself is nonetheless associated with a higher share of specialists (−0.150) and is the next strongest coefficient to the number of hospital beds. An increase of beds by two standard deviations from the mean shows a decrease in the GP-specialist ratio of −0.206. Weaker effects increasing the share of specialists can be found with a higher migration balance of the last 5 years (−0.113), a higher rate of highly qualified workers (−0.099), a higher household income (−0.088), and share of privately insured persons (−0.063).

The only two variables associated with a higher share of GPs when positively deviating from the mean are the travel time to middle-order centers (0.115) and the number of middle-order centers (0.052). The only ASHIP showing a significant difference in the GP-to-specialist ratio is Bavaria with a higher share in GPs by 0.187.

## Discussion

### Interpretation of results

The present study analyzes regional differences in the density of general practitioners, specialists, and the ratio of GPs divided by specialists using a zero-truncated negative-binomial linear model. Physician density was estimated over independent variables concerning the 412 districts in Germany controlling for the different spheres of responsibility of the 17 ASHIPs regulating the distribution of physicians. For all three analyses, several models have been estimated, and variables were kept in the model if they showed statistical significance at the 0.05 level in any of the three models.

The respective densities of GPs and specialists show strong associations with the population density of a district. While the results from the specialist model are in line with the prediction of our theoretical model suggesting a positive correlation with the population density, GP density seems to be negatively associated with population density contradicting the theoretical conclusions.

In all models, significant interactions are changing the coefficient of the population density substantially. At first glance, the presence of a city seems to reverse the respective association between population density and physician density. Accordingly, these findings might suggest that specialist density and the GP-specialist ratio increase and GP density decreases with increasing population density until some level of urbanization is reached. This may be due to a historically grown higher density in urban areas which has been capped by the needs-based planning mechanism since 1995 or the saturation of patient demand. Furthermore, the effects of some variables from the supply side might be correlated with population density, e.g., urban areas tend to have more labor in the tertiary sector requiring highly qualified workers or having a higher household income.

Surprisingly, the old-age dependency ratio, life expectancy, and mortality as proxies for morbidity of the inhabitants are not significantly correlated with the density of GPs or specialists for data in 2010. This might be due to governmental regulations not incorporating the population age structure as a proxy for morbidity in the needs-based planning until 2012 [46]. The proxies for financial incentives only play a minor role as indicated by the effects of household income and the share of privately insured persons.

As can be seen from the coefficients of the supply-side variables, regional preferences in terms of our theoretical model take effect beside pure income maximization, i.e., this means that *w* > 1. For example, there is an increasing effect of the cultural variables, clearly exemplified by the specialist model, where the strong effect of state capitals may be due to state-operas, -theaters, or -museums. A higher touristic attractiveness of a district seems to indicate a higher number of GPs. However, some coefficients seem to be counterintuitive as travel time to the nearest airport in the first analysis and unemployment in the first and second model are positive. This means that a shorter travel time to the nearest airport and a lower rate of unemployment are associated with a lower density of physicians. While the travel time might be overruled by other variables also covering airports, e.g., the number of high-order centers or the touristic attractiveness of a district, there might be another explanation for the unemployment rate than being an artifact. As there is evidence that unemployment causes negative effects on mental [47] and physical health [48], the unemployment rate may serve as a proxy for morbidity.

The effect size of the coefficients needs to be considered carefully as the variables are standardized, and the coefficients indicate the change in the dependent variable if the independent variable is changed by two standard deviations from the mean. For example, as the minimum and maximum of the rate of privately insured are only 1.1 and 1.6 double-standard deviations from the mean, the full coefficient will not be used in the sample. In contrast, the maximal touristic attractiveness lies 5.6 times from the mean allowing for a greater overall effect of this variable.

In summary, the findings therefore provide some evidence that the first hypothesis derived from the theoretical model that a higher density of physicians can be found in districts with a higher population density. As we have controlled for factors which are associated with physicians’ regional preferences, a relevant part of physician density can be solely explained by patient demand differences.

Compared to the number of GPs, the density of specialists is generally more strongly associated with the population density and other variables describing an urbanized district. For example, a higher household income and a higher travel time to the next middle-order center indicate a higher density of specialists in comparison to the population parameters alone. These results underscore the second hypothesis of the theoretical model that a higher density of specialists might be even be more associated with urban districts than is the case of the density of GPs.

Furthermore, the third model analyzed the ratio between GPs and specialists. The negative association between this ratio and the population density supports hypothesis number 3 from our theoretical model. Nevertheless, it must be stated that in this model as well the interaction term weakens the effect of the population density in urbanized areas. However, there still remains a considerable effect, and other variables contribute to the effect of population density on the ratio between GPs and specialists.

### Limitations

There are also a number of limitations that need to be considered. First of all, not all the data used in the model were available for the same year. However, the maximum difference is 1 year, and it is assumed that no drastic changes of the independent variables occurred within this short period of time. Secondly, districts are politically administered borders leading to willfully varying district sizes in the different states. Therefore, homogeneity of the explaining variables may be smaller in larger districts reducing effect sizes and leading to an underestimation of the coefficient sizes. In addition, the number of physicians and the share of privately insured patients in each district could not be retrieved from official statistics. This makes it hard to validate or to reconstruct the data, as, for example, the dataset of privately insured persons is based on nearly 25% missing values. With the number of physicians and a dummy variable for eastern Germany as part of the predictive variables used in the multiple imputation equations, it may be that the number of GPs and specialists is—to a large part—explained by the overall number of physicians [39].

Furthermore, the model did not consider random slopes, although the number of physicians is regulated by 17 independent ASHIPs. The varying intercept model was chosen, as only minor differences in the criteria for the distribution of physicians between the ASHIPs are allowed by the planning regulations [26]. Nevertheless, there might be differences in budget ceilings and different degrees to which some services are reimbursed between the ASHIPs. As data on this topic is not openly available, it was not possible to control for those factors. This is also true for factor of referrals between GPs and specialists which might also effect the practice location decision of a physician.

In this context, it should be mentioned that it might also not be appropriate to assume linear effects of the variables, thus ignoring saturation effects. Furthermore, as the analyses were carried out using cross-sectional data, no causal interpretations can be made. On the other hand, the data used in this paper represent a complete survey of German outpatient physicians and facilitate reflecting the differences between the 17 ASHIPs responsible for the allocation of physicians’ practices.

## Conclusions

Differences in regional specialist supply can to some extent be explained by differences in population density. Other variables beyond the number of potential patients and their demographic structure have influence on the supply and demand side of the regional health care markets. This might have important policy implications in terms of the future selection of variables (like the unemployment rate or the number of hospital beds) to determine the medical need and possible undersupply by governmental regulation bodies in a particular area.

## Endnote

^a^According to the definition of the planning regulations, specialists are ophthalmologists, surgeons, gynecologists, dermatologists, otolaryngologists, neurologists, orthopedists, psychotherapists, urologists, and pediatricians [46].

## Additional file

Additional file 1:
**Diagnostics of empirical models and intercepts of the different ASHIPs.** The file contains a diagnostics GP-density model, diagnostics specialist-density model, and a diagnostics ratio model. All intercepts of ASHIPs in the states of former East Germany have a negative association with the ratio of GPs to specialists. Only the ASHIP regions of the former West German states Westphalia and Saarland also show negative correlations.
