# Supplementary material for: Regional differences of outpatient physician supply as a theoretical economic and empirical generalized linear model
Source: Hum Resour Health. 2015 Nov 17;13:85. doi: 10.1186/s12960-015-0088-1 (PMC4647336; doi:10.1186/s12960-015-0088-1)
Supplement: Additional file 1: — Diagnostics of empirical models and intercepts of the different ASHIPs. The file contains a diagnostics GP-density model, diagnostics specialist-density model, and a diagnostics ratio model. All intercepts of ASHIPs in the states of former East Germany have a negative association with the ratio of GPs to specialists. Only the ASHIP regions of the former West German states Westphalia and Saarland also show negative correlations. [file 12960_2015_88_MOESM1_ESM.docx]

**Appendix for Reviewer: Diagnostics of empirical models**


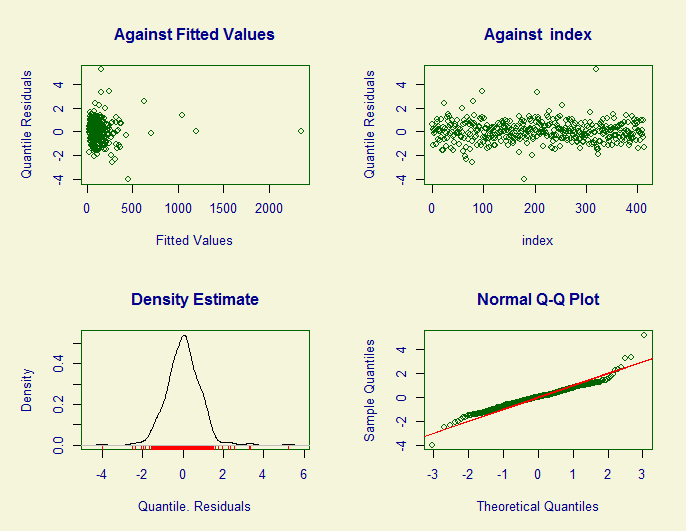


Figure 1: Diagnostics GP-density model.


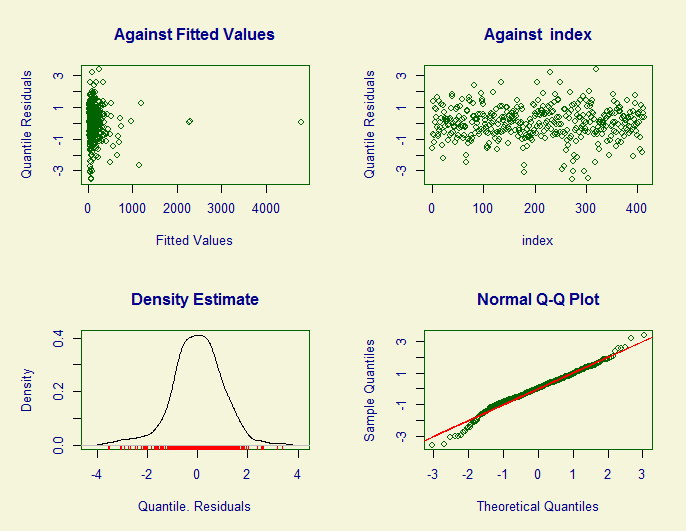


Figure 2: Diagnostics specialists-density model.


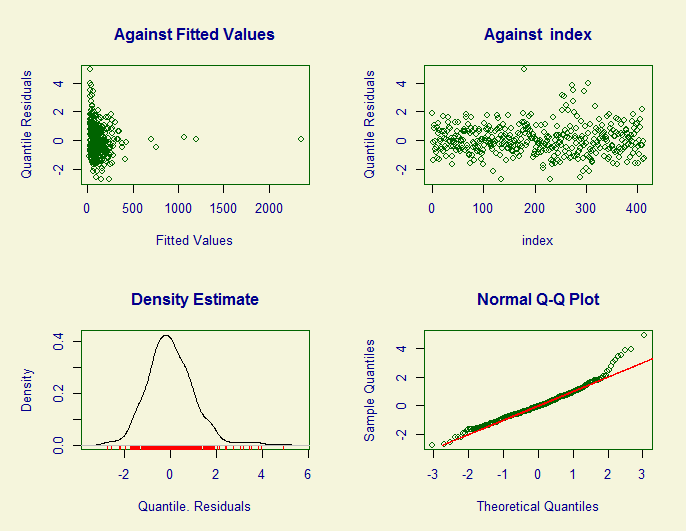


Figure 3: Diagnostics ratio model.

**Intercepts of the different ASHIPs**

As can be seen in the below table A1, all intercepts of ASHIPs in the states of former East Germany (marked by E) have a negative association with the ratio of GPs to specialists. Only the ASHIP regions of the former West German (marked by E) states Westphalia and Saarland also show negative correlations.

Table A1: Varying intercepts of the three zero-truncated, negative binomial GLMs for GP density, specialists density and ratio of GPs divided by specialists.

|  | **General physicians** | | **Specialists** | | **Ratio GPs/specialists** | |
| --- | --- | --- | --- | --- | --- | --- |
| **ASHIP** | **Estimate** | **p-value** | **Estimate** | **p-value** | **Estimate** | **p-value** |
| Brandenburg (E) | Reference | | | | | |
| Berlin (W) | 0.217 | 0.000*** | 0.382 | 0.035* | -0.152 | 0.381 |
| Baden Württemberg (W) | 0.103 | 0.014* | 0.072 | 0.346 | 0.061 | 0.406 |
| Bavaria (W) | 0.193 | 0.000*** | 0.059 | 0.452 | 0.187 | 0.013* |
| Bremen (W) | 0.122 | 0.049* | 0.148 | 0.260 | -0.113 | 0.393 |
| Hessen (W) | 0.062 | 0.134 | 0.156 | 0.037* | -0.066 | 0.366 |
| Hamburg (W) | 0.173 | 0.002** | 0.138 | 0.434 | 0.056 | 0.738 |
| Mecklenburg-  Western Pomerania (E) | 0.064 | 0.154 | -0.020 | 0.779 | 0.074 | 0.294 |
| Niederrhein (W) | 0.082 | 0.036* | 0.019 | 0.795 | 0.085 | 0.226 |
| Lower Saxony (W) | 0.038 | 0.293 | 0.102 | 0.113 | -0.057 | 0.357 |
| Rhineland-  Palatinate(W) | 0.098 | 0.022* | 0.028 | 0.713 | 0.088 | 0.227 |
| Saxony Anhalt (E) | 0.030 | 0.445 | 0.027 | 0.692 | -0.025 | 0.705 |
| Schleswig-Holstein (W) | 0.149 | 0.000*** | 0.095 | 0.221 | 0.068 | 0.361 |
| Saarland (W) | 0.237 | 0.000*** | 0.234 | 0.019* | -0.030 | 0.755 |
| Saxony (E) | 0.070 | 0.045* | 0.124 | 0.068 | -0.048 | 0.460 |
| Thuringia (E) | 0.074 | 0.072 | 0.047 | 0.489 | 0.028 | 0.670 |
| Westfalia-Lippe (W) | 0.006 | 0.883 | -0.038 | 0.595 | 0.059 | 0.391 |
